# Successful Decoding of Famous Faces in the Fusiform Face Area

**DOI:** 10.1371/journal.pone.0117126

**Published:** 2015-02-25

**Authors:** Vadim Axelrod, Galit Yovel

**Affiliations:** 1 School of Psychological Sciences, Tel Aviv University, Tel Aviv, Israel; 2 School of Neuroscience, Tel Aviv University, Tel Aviv, Israel; University of British Columbia, CANADA

## Abstract

What are the neural mechanisms of face recognition? It is believed that the network of face-selective areas, which spans the occipital, temporal, and frontal cortices, is important in face recognition. A number of previous studies indeed reported that face identity could be discriminated based on patterns of multivoxel activity in the fusiform face area and the anterior temporal lobe. However, given the difficulty in localizing the face-selective area in the anterior temporal lobe, its role in face recognition is still unknown. Furthermore, previous studies limited their analysis to occipito-temporal regions without testing identity decoding in more anterior face-selective regions, such as the amygdala and prefrontal cortex. In the current high-resolution functional Magnetic Resonance Imaging study, we systematically examined the decoding of the identity of famous faces in the temporo-frontal network of face-selective and adjacent non-face-selective regions. A special focus has been put on the face-area in the anterior temporal lobe, which was reliably localized using an optimized scanning protocol. We found that face-identity could be discriminated above chance level only in the fusiform face area. Our results corroborate the role of the fusiform face area in face recognition. Future studies are needed to further explore the role of the more recently discovered anterior face-selective areas in face recognition.

## Introduction

Faces are recognized quickly and effortlessly. Over the past two decades, the underlying neural mechanisms of face processing have become gradually elucidated. In particular, a highly established finding in neuroimaging studies is the occipito-temporal network of face-selective regions: occipital face area (OFA) [1], the fusiform face area (FFA) [2] and the posterior superior temporal sulcus (pSTS)[3]. More recently, three more anterior regions have been reported to show face-selective responses. This includes face-selective areas in the anterior temporal lobe (ATL) [4,5], the amygdala [6–9] and the prefrontal cortex [4,9,10]. Notably, whereas all these regions clearly show a higher response to faces than non-face stimuli, their role in discriminating between different face identities is still unclear.

To date, although no clear picture has emerged, a number of imaging studies have suggested that the FFA [11–15] (but see: [16–19]) and the ATL [11,13,14,19–25] might play a role in face recognition. However, empirical evidence regarding the ATL is especially complex. In particular, while the face-selective ATL was implicated in face recognition [4,5,20,21,26–28], the face-selectivity (higher response to face than to non-faces) of the clusters in the ATL that discriminated between face identities has not been tested [11,13–15,19]. This discrepancy might stem from severe magnetic susceptibility artifacts in the ATL (e.g., [29,30]) and consequently low reliability of the face-selective responses reported in this area. In contrast to the unclear findings with respect to the processing of face identity in the human face-selective ATL, studies of the monkey face-selective areas have shown clear identity selective view-invariant tuning in the most anterior face patch—AM [27]—which may be the human homologue of the face-selective area in the anterior temporal lobe [31]. Finally, previous studies have focused only on occipital and ventral temporal regions, while the role of additional anterior face-selective regions in face recognition, such as the amygdala and prefrontal cortex, has not been tested.

**Fig 1:**
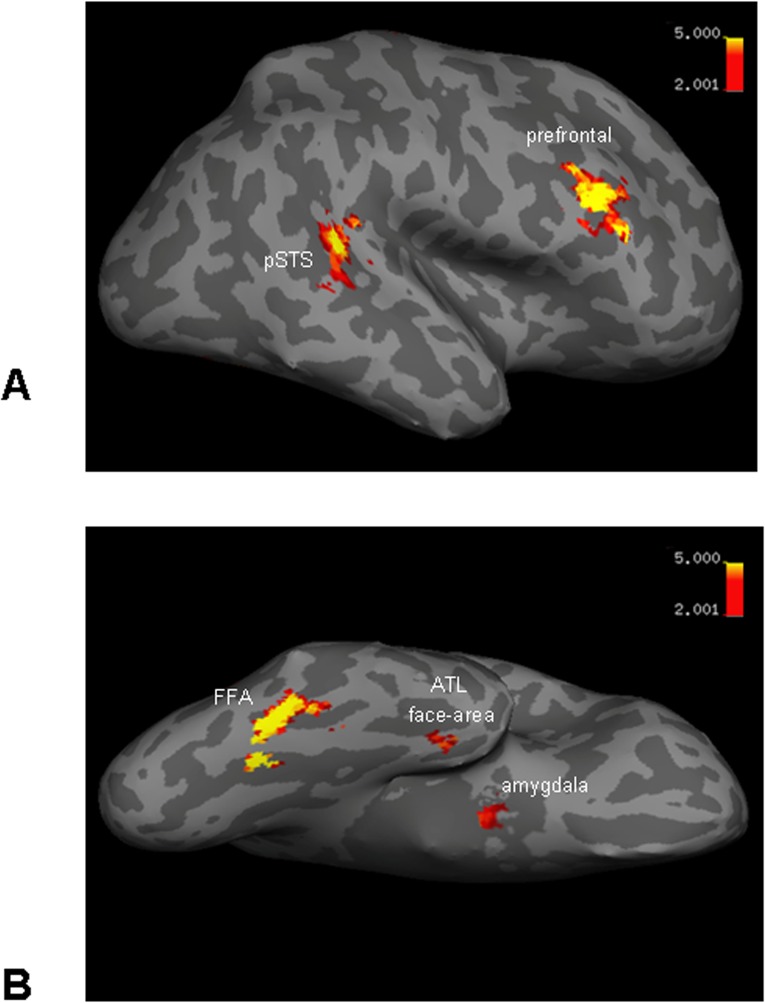
Face-selective areas of one representative participant (inflated cortex, right hemisphere). (A) Lateral brain view: posterior STS and prefrontal face-selective areas. (B) Ventral brain view: FFA, ATL face-area and amygdala face-selective areas.

In the current high-resolution fMRI study, we systematically explored the role of temporo-frontal face-selective regions (FFA, posterior superior temporal sulcus (STS), ATL face-area, amygdala, and prefrontal cortex; Fig. 1) in recognition of famous faces. Participants were presented with 8 different images of each of two famous faces. We conducted Region of Interest (ROI) Univariate and Multivoxel Pattern Analysis (MVPA) [32] to determine the role of face-selective and adjacent non-face areas in discriminating between these two famous identities. A special emphasis was put on the ATL face-area, which was reliably localized using recently proposed scanning optimization [9]. In addition, in contrast to previous identity decoding studies that used unfamiliar faces [11,13,14,19], we used familiar (famous) identities as they 1) are better discriminated behaviorally [33,34]; 2) exhibit enhanced invariant neural face representation (e.g., view-invariance) [35–37]; and 3) are more likely to be discriminated in the ATL, given that the ATL is known to be involved in semantic processing [38].

## Materials and Methods

### Apparatus

MRI data were collected using a 3T GE MRI scanner with an 8-channel head coil. Echo planar imaging sequence includes the following parameters: TR = 2 sec, TE = 30 ms, flip angle: 90, slice thickness: 2.4 mm no gap, FOV 200 mm, data was acquired using 96x96 matrix (in plane resolution 2.08x2.08 mm), reconstruct into 128x128 matrix (in plane resolution 1.56x1.56 mm). Slices orientation was coronal, parallel to brain stem [9]. Number of slices was varied between participants from 23 to 25 slices (the maximal number of slices per participant according to slice orientation described above). The scanning was executed using a multislab method [39], where first slab comprised of 11 slices and covered anterior temporal region ventrally with parts of the frontal lobes dorsally. The remaining 12–14 slices covered mid-temporal areas ventrally and part of the parietal lobe dorsally. The coronal slice prescription has been shown to improve magnetic susceptibly in the ATL [9]. Anatomical SPGR images (full brain coverage) were collected with 1x1x1 mm resolution (TE = 3.52 ms, TR = 9.104 ms). LCD projector (NEC, VT660K) was used for projecting the stimuli. Projector was positioned ahead of the participant and the stimuli were viewed through a tilted mirror mounted on the MR head coil. Fiber-optic MR-compatible response box (Current Designs, Philadelphia, PA) has been used to register behavioral responses during scanning.

### Participants

18 healthy volunteers (age: 18–40, 11 females, all right-handed) participated in the experiment. One participant was excluded from the analysis due to excessive movements in the scanner (>1 cm). All participants gave informed consent to participate in the study. The study was approved by the ethics committee of the Tel Aviv Sourasky Medical Center. Participants provided written informed consent to participate in this study. Data from 9 participants was also used in different analysis in our recent publication [9].

### Experimental Stimuli

**Fig 2:**
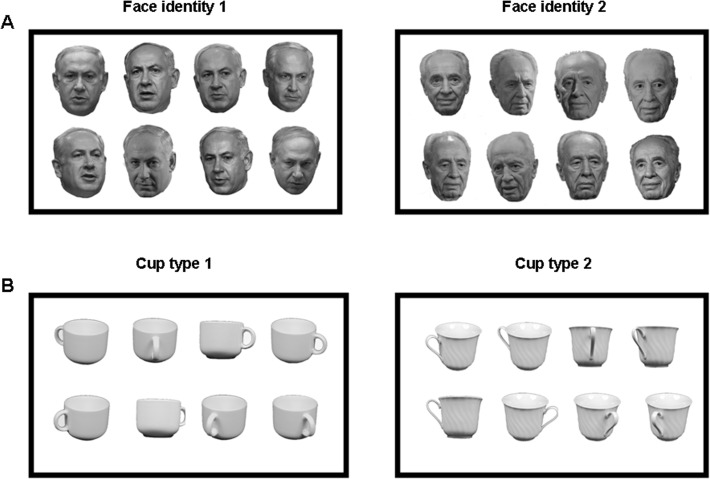
The face and cup stimuli used in the experiment. (A) Eight images of Benjamin Netanyahu (the prime minister of Israel) and eight images of Shimon Peres (the president of Israel). (B) Eight images of two cup types.

The experiment included images of two categories: faces and cups. All the images were grey-scaled. The faces were of two Israeli highly familiar politicians: Benjamin Netanyahu (the prime minister of Israel) and Shimon Peres (the president of Israel) (Fig. 2A); the cups were of two different types (Fig. 2B). We verified with each of the participants before including them in the experiment that they are familiar with the two famous faces included in the experiment. Each face identity/cup type was represented by 8 different images. The face pictures were taken on different occasions mostly from front view (up to ∼10° of view angle rotation) with neutral expression. The cups were pictures taken from different views. Face images were collected from various Internet sources. Images of cups were from ETH80 dataset (https://www.d2.mpi-inf.mpg.de/Datasets/ETH80) [40]. The pictures of faces and cups were first cropped from the background. Then luminance and color between all images was adjusted using “match color” function of Photoshop CS2. Finally, a white monotonic background was added to all images. The size of all stimuli was 7x7 degrees of visual angle.

To test for low-level differences between stimuli we conducted image similarity analysis. We first examined whether there are any systematic low-level differences between the two sets of images of two face identities. To that extent, we calculated image pixel correlation (e.g., [41,42]). First, for each face identity image set, we calculated pixel correlation within the image set (28 image pairs for each set). Average pixel correlation within face identity image set 1 was 0.817 (MSE: 0.005) and within face identity image set 2 was 0.828 (MSE: 0.007). The difference between the pixel correlation of two image sets was insignificant (two-tailed t-test: t(54) = 1.23, p = 0.22). Next, we calculated pixel correlation across identity image sets (64 image pairs). The correlation was 0.813 (MSE: 0.004). Critically, there was an insignificant difference for pixel correlation between and within image sets (identity 1 vs. between identities: t(66) < 1; identity 2 vs. between identities: t(47) = 1.69, p = 0.096; two-tailed t-test with unequal variance [43]). Taken together, we conclude that there is no evidence that two sets of identities differ in their low-level image-based properties.

The same analysis was conducted for two image sets of cup stimuli. Average pixel correlation within cup type 1 was 0.692 (MSE: 0.022) and within cup type 2 was 0.70 (MSE: 0.018). The difference between the pixel correlation of the two image sets was insignificant (two-tailed t-test: t(54) < 1). Pixel correlation across cup image sets (64 image pairs) was 0.64 (MSE: 0.008). The pixel correlation between image sets was significantly lower than within image sets (cup 1 vs. between cup types: t(35) = 2.02, p = 0.05; cup 2 vs. between cup types: t(39) = 2.79, p = 0.008). Thus, we conclude that two types of cups differed in their low-level image-based properties. Notably, discrimination between the two types of cups was not the main focus of the present study.

### Experimental Design

The stimuli were presented in blocks (block-design). The blocks were of 4 types: two face identities and two cup types. Each block lasted 16 seconds and comprised of 16 images. Each image was presented for 0.3 seconds and the inter-stimulus interval time was 0.7 seconds. To avoid consecutive presentation of two blocks of the same category, blocks within each session were arranged in triplets of either faces-cups-fixation or cups-faces-fixation. The duration of fixation block was 8 seconds. In each session there were 10 face blocks (5 for each identity), 10 cup blocks (5 for each cup type) and 10 fixation blocks. The order of face and cup blocks was counterbalanced. To ensure that the participants pay attention to the stimuli, they were asked to press a response key whenever the same image appeared in two consecutive trials (one-back task). The number of target trials varied from block to block (minimum: 0, maximum: 4). To prevent discrimination based on apparent motion, the location of the stimuli varied across trials with a random jitter of 20 pixels. The total session duration was 6:52 min. Fourteen participants completed 5 sessions and three participants completed 6 sessions.

### Data Analysis

**Preprocessing.** SPM5 (Wellcome Trust Centre for Neuroimaging, London, UK; http://www.fil.ion.ucl.ac.uk) was used for data analysis. The functional scans were realigned, motion corrected, normalized to 2x2x2 voxel resolution using MNI template and smoothed with a FWHM = 3 mm kernel. The normalization was done using unified segmentation procedure [44].

**Region of Interest independent localization.** Regions of interest were localized using first session (10 blocks of faces and 10 blocks of cups). Face identity and cup type main discrimination analysis was done using all other sessions (see below). For Region of Interest (ROI) localization we estimated GLM model (HRF boxcar function) with two regressors: faces and cups. For defining face-selective regions the faces > cups contrast was used. The face-selective FFA, pSTS, prefrontal cortex were defined using p<0.001, uncorrected threshold and ATL face-area and amygdala were defined using p<0.01, uncorrected threshold. Non-face selective region in the collateral sulcus was defined using cups > faces contrast (p<0.001, uncorrected) [45]. ROIs definition was done using the MarsBaR region of interest toolbox for SPM [46]. Cortical reconstruction of representative participant (inflated cortex map in Fig. 1) was created using Freesurfer image analysis 4.5 (http://surfer.nmr.mgh.harvard.edu/) [47].

**Fig 3:**
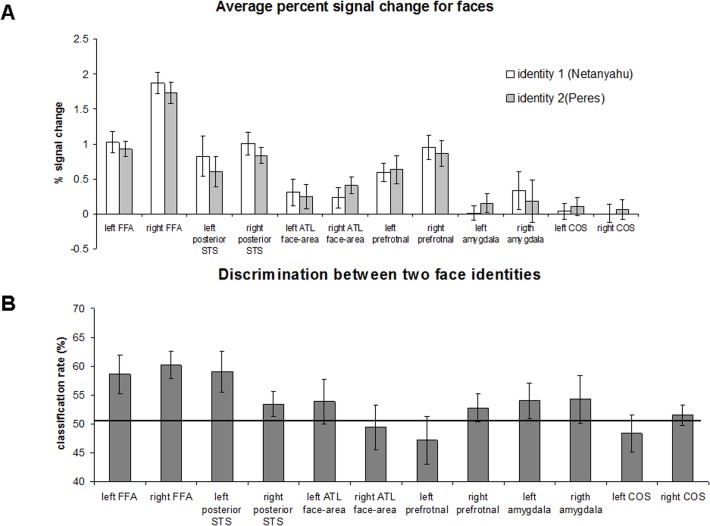
Region of Interest-based face identity discrimination analysis (Benjamin Netanyahu and Shimon Peres identities). (A) Average percent signal change for two face identities in the different face-selective areas and non-face selective collateral sulcus area. Error bars denote standard error of the mean. (B) Classification rates between face identities in face and non-face selective regions. The black line indicates a chance level of 50%. The error bars denote the standard error of the mean.

**Fig 4:**
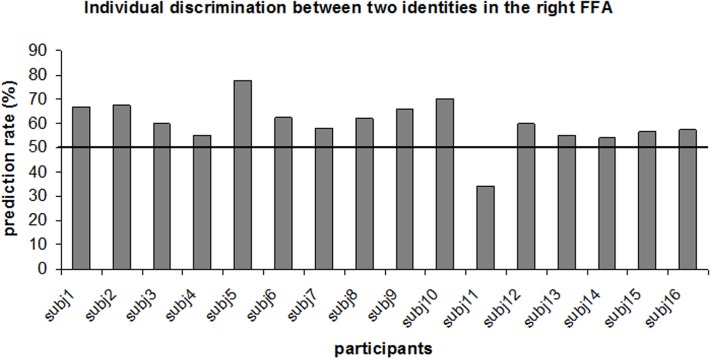
Individual classification rates of identity discrimination analysis in the right FFA.

**Fig 5:**
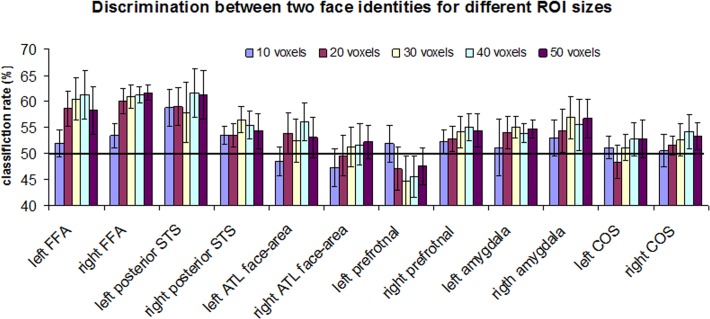
Classification rates in face-selective (FFA, pSTS, ATL face-area, prefrontal face-area, amygdala) and non-face selective collateral sulcus area for different ROI sizes (10, 20, 30, 40 and 50 voxels). The black line indicates a chance level of 50%. The error bars denote the standard error of the mean.

The functional and anatomical ROIs, defined in the previous step had different sizes. It has been previously shown that classification rate might be influenced by the ROI size [42,48–50]. Therefore, it was important to use the same number of voxels for the ROIs of different regions. For the main discrimination analysis (Fig. 3 and Fig. 4) the ROI comprised of 20 voxels (160 mm^3^), while they were selected as a contiguous cluster of most selective voxels (e.g., [50–52]). Number of participants per region, average z-score and average MNI coordinate is shown in Table 1. For the discrimination analysis with different ROI size (Fig. 5) the most active voxels with the following size were selected: 10 voxels (80 mm^3^), 30 voxels (240 mm^3^), 40 voxels (320 mm^3^) and 50 voxels (400 mm^3^). For the control analysis we also conducted discrimination using ROIs of full size, before equalization procedure.

**Table 1:** Specification of face-selective and object-selective regions of interest (ROIs) used in the main analysis: number of subjects, average ROI z-score contrast value, and MNI coordinates of the localized.

ROIs	number of participants	average z-score	MNI coordinates		
			X	Y	Z
**left FFA**	13	7.03	-41	-53	-18
**right FFA**	16	8.50	39	-52	-16
**left pSTS face area**	8	5.6	-51	-54	11
**right pSTS face area**	12	6.5	52	-42	10
**left ATL face area**	7	3.16	-33	-16	-37
**right ATL face area**	11	3.51	36	-11	-40
**left prefrontal face area**	8	4.18	-43	19	27
**right prefrontal face area**	13	5	44	19	26
**left amygdala face area**	8	2.88	-19	-3	-16
**right amygdala face area**	8	3.43	21	-3	-17
**left COS object area**	9	-4.59	-29	-55	-11
**right COS object area**	12	-4.39	29	-52	-10

Size of all ROIs was 20 voxels (160 mm^3^).

**Face identities discrimination analysis.** The data in this analysis included all sessions except for the first session, which was used for the functional localization.

### Univariate analysis

**Fig 6:**
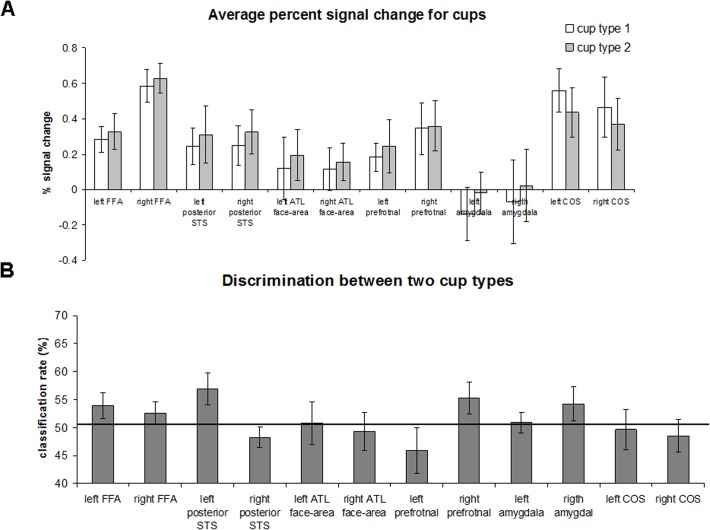
Region of Interest discrimination analysis for cups. (A) Average percent signal change for two cup types in the face-selective areas and non-face selective collateral sulcus area. Error bars denote standard error of the mean. (B) Classification rates between two cup types in face and non-face selective regions. The black line indicates a chance level of 50%. The error bars denote the standard error of the mean.

For the univariate analysis, we estimated GLM model (HRF boxcar function) with four regressors: face identities 1,2 and cup types 1,2. This model was used to calculate percent signal change for each condition (for each face identity or cup type) within the predefined ROIs (Fig. 3A and Fig. 6A). Time courses were extracted for each of four regressors (identity 1,2 and cup type 1,2). Block plateau values (from TR = 4 to TR = 10 from block onset) were averaged and submitted to paired t-test analysis (SPSS 17). Time courses were extracted using the MarsBaR region of interest toolbox for SPM [46].

### Multivoxel ROI pattern analysis

The Multivoxel Pattern Classification analysis procedure was similar to what was used in our previous studies [50,52]. Raw intensity values were used for pattern classification analysis. In the multivariate analysis we also used normalized data because 1) this allowed us to compare between univariate and multivariate results; 2) our preliminary data analyses show that there is no benefit for using non-normalized data for classification. The pattern classification data was detrended and normalized using z-score MATLAB function [53]. This procedure was applied for the full session voxel time course (for each session separately). In addition, the time course was shifted two TRs (4 seconds) to account for hemodynamic lag. For each of the four classes (two face identities and two cup types), the mean intensity (global signal) for the condition was subtracted from the voxel intensity value [54]. This procedure was performed separately for the data from each session so no information leakage occurs in a cross-validation procedure. Subtraction of the global average has been previously suggested to improve multivoxel prediction [55]. Each session consisted of 5 blocks per class, with 8 TRs per block. For each experimental block the preceding block could be either fixation or experimental block, which created a contamination of the beginning of the block [56]. Our preliminary pilots showed that first two volumes of the block were the most contaminated and therefore they were discarded. The remaining 6 TRs data points were averaged resulting in a single data point per block. The leave-one-session-out cross-validation procedure was repeated according to number of sessions completed by subject. The classification package was the LibSVM MATLAB implementation of the linear support vector machine (http://www.csie.ntu.edu.tw/∼cjlin/libsvm/). The pattern classification analysis was performed using a custom made MATLAB code [53]. Significance of the classification results was established as a group level one-tail t-test above the chance of the individual classification rates [14,42,57] with Bonferroni correction for multiple comparisons according to the number of regions: p = 0.05/12 = 0.004.

**Multivoxel search-light pattern analysis in the ATL.** Analysis was limited to the ATL region. The brain volume used in the analysis was defined as scanned volume that was in overlap with Brodmann Areas 20 and 38 (http://fmri.wfubmc.edu/software/PickAtlas [58]). Notably, anterior portion of the ATL (approximately more anterior MNI Y coordinate = 0) has not been included in the analysis as it has not been covered with our slice prescription. The search-light size was 27-voxel size (3x3x3 voxels box = 216 mm^3^). Classification was performed on individual subject data. The search-light was moved iteratively over the ATL with 1 voxel step each time. In each step a search-light classification rate was assigned to all the voxels, which were included in this search-light. At the end of whole process, each voxel classification result was a vector of classification rates of all the search-lights it was included in. The final voxel classification rate was the average of this vector. This search-light method is similar to "Monte Carlo sampling search-light" approach [59], which was shown to result in a higher discriminative power comparing to a strategy of assigning prediction rate to a central voxel of the search-light box (or sphere). The statistical significance was assessed as one-tailed t-test against chance level (0.5) for each voxel across all participants with p<0.05, false discovery rate (FDR) correction [14]. As a control analysis, we also conducted a search-light analysis with non-overlapping search-lights (step of 3 voxels), where each voxel was classified only once.

### Replication experiment with a different set of famous faces

The face stimuli we used in the functional localizer and the identity discrimination experiment were the same. To find out whether the results we revealed will be replicated also when a standard face localizer and a different set of famous faces are used in the decoding task, 5 participants (3 females, average age: 27.5) returned to participate in a replication experiment. The experiment was conducted 3.5 years after the original experiment and used exactly the same methods, except for the differences specified below.

**Stimuli.** The face stimuli in the identity discrimination experiment were of two highly familiar actors: Leonardo DiCaprio and Brad Pitt. Following the logic of the original experiment, the face identities of two actors were chosen because they belong to the same semantic category. All the participants were familiar with both faces. As in the original experiment, each face identity was represented by 8 different images (up to ∼10° of view angle rotation; neutral face expression). Image preprocessing steps were the same as in the original experiment. The size of all stimuli was 7x7 degrees of visual angle.

To test for low-level differences between stimuli of famous faces we conducted image similarity analysis. Average pixel correlation within face identity 1 was 0.834 (MSE: 0.006) and face identity 2 was 0.837 (MSE: 0.008). The difference between the pixel correlation of the two image sets was insignificant (two-tailed t-test: t(54) < 1). Pixel correlation across identity image sets (64 image pairs) was 0.824 (MSE: 0.005). Critically, there was no significant difference for pixel correlation between and within image sets (identity 1 vs. between identities: t(65) = 1.21, p = 0.22; identity 2 vs. between identities: t(54) = 1.32, p = 0.19; two-tailed t-test with unequal variance [43]). Thus, there is no evidence that two sets of identities differ in their low-level image-based properties.

**Experimental Design.** The face-selective and object-selective regions were localized using standard independent functional localizer that included various images of unfamiliar faces and objects [9,52,60,61]. The design of the functional localizer experiment was exactly the same as the original experiment (10 blocks per condition/session). The participants underwent two sessions of the functional localizer.

Design of the identity discrimination experimental sessions was the same as the original experiment. Three participants completed 5 sessions and two participants completed 6 sessions. Sessions of the identity discrimination experiment were interleaved with functional localizer sessions.

## Results

### Behavioral Results

Behavioral results on the one-back task in the scanner were high under all conditions: 92% for face identity 1, 89% for face identity 2, 94% for cup type 1, 90% for cup type 2. No significant difference in performance was found between the two face identities and two cups (t(17) < 1). Thus, task difficulty did not differ between face identities and cup types.

### Neuroimaging Results

In order to obtain a better signal in the ATL face-area we used an optimized scanning sequence proposed recently [9]. In particular, we used a coronal slice orientation with two slabs that covered anterior temporal region ventrally with parts of the frontal lobes dorsally (anterior slab) and mid-temporal areas ventrally with part of the parietal lobe dorsally (posterior slab). This slice prescription permitted to cover the whole network of face-selective regions, except for the occipital face area (OFA). Face-selective regions of one representative participants are shown in Fig. 1. In Table 1, we show ROI summary statistics of the face-selective regions (FFA, pSTS, ATL face-area, prefrontal and amygdala) and non-face-selective region in the collateral sulcus (COS) defined by voxels showing higher response to cups than faces. The regions were localized independently, using the first session of the experiment (see Methods).

First, we first tested whether, based on average fMRI signal, it is possible to discriminate between the two face identities. The results are shown in Fig. 3A. No significant difference in activation between the two identities was found in any of the regions (paired, two-tailed t-test: left FFA: t(12)<1; right FFA: t(15) = 1.26, p = 0.22; left pSTS: t(7) = 1.47, p = 0.19; right pSTS: t(12) = 1.29, p = 0.21; left ATL face-area: t(6)<1, right ATL face-area: t(10) = -1.89, p = 0.09; left prefrontal: t(7) = -1.32, p = 0.22; right prefrontal: t(12)<1; left amygdala: t(7)<1; right amygdala: t(7) = 1.88, p = 0.1; left COS: t(8) = -1.31, p = 0.22; right COS: t(11) = -1.48, p = 0.16).

Next, we examined whether using multivoxel pattern analyses (MVPA) we could correctly decode face identity. To ensure that any difference in the global level of activation between identities does not influence the MVPA results, the global signal level was subtracted prior to classification analysis separately for each condition [54]. The results of the MVPA are shown in Fig. 3B. Statistical significance was assessed using one-tailed t-test against the chance level of 0.5 (Bonferroni multiple comparison correction for number of regions (n = 12): p<0.004). The prediction rate was significantly above chance in the right FFA (t(15) = 4.29, p<0.001). In the left FFA and the left pSTS, the prediction rate was greater than chance; however, it did not reach statistical significance after multiple comparison correction: left FFA (t(12) = 2.57, p = 0.012), left pSTS (t(7) = 2.54, p = 0.019). In other regions, the prediction rate did not differ significantly from chance level: right pSTS: t(12) = 1.53, p = 0.08; left ATL face-area: t(6)<1; right ATL face-area: t(10)<1; left prefrontal: t(7)<1; right prefrontal: t(12) = 1.16, p = 0.13; left amygdala: t(7) = 1.31, p = 0.11; right amygdala: t(7)<1; left COS: t(8) = <1; right COS: t(11)<1. Notably, it could be claimed that the high significance achieved in the right FFA, but not in other regions, might be a consequence of the largest number of ROIs in this region (Table 1). To address this point, in Fig. 4, we show individual prediction rates in the right FFA. As can be seen, for all the participants except for one, predictions were above chance level (50%). Thus, highly significant prediction result in the right FFA cannot be explained solely by large number of ROIs.

The results reported thus far were based on an ROI size of 20 voxels (160 mm^3^). To test the reliability of successful prediction in the FFA, we repeated the analysis for several ROI sizes: 10 voxels (80 mm^3^), 30 voxels (240 mm^3^), 40 voxels (320 mm^3^) and 50 voxels (400 mm^3^). The results of this analysis are shown in Fig. 5 and Table 2. As can be seen, for ROI larger than 10 voxels, the right FFA was the only region where two face identities could be consistently decoded above chance level (after multiple comparison correction). Finally, we conducted a control analysis where discrimination between two identities was tested using ROIs that were defined to include all the voxels that passed functional localization threshold (no ROI equalization procedure; see Methods). The results were qualitatively similar to what we reported for equalized size ROIs. The discrimination between two identities was significantly above chance in the right FFA (t(15) = 3.44, p = 0.0018). In the left FFA, the left pSTS and right prefrontal, the prediction rate was greater than chance; however, it did not reach statistical significance after multiple comparison correction: left FFA (t(12) = 2.44, p = 0.015], left pSTS (t(7) = 3.1, p = 0.008), right prefrontal (t(12) = 2.65, p = 0.01). In other regions, the prediction rate did not differ significantly from chance level: right pSTS: t(12) = 1.69, p = 0.058; left ATL face-area: t(6)<1; right ATL face-area: t(10)<1; left prefrontal: t(7)<1; left amygdala: t(7) = 1.85, p = 0.053; right amygdala: t(7) = 1.67, p = 0.069; left COS: t(8) = <1; right COS: t(11)<1.

**Table 2:** Results of discrimination between two face identities (MVPA analysis).

Region	10 voxels	20 voxels	30 voxels	40 voxels	50 voxels
**left FFA**	t(12)<1	t(12) = 2.57,p = 0.01	t(12) = 2.61,p = 0.011	t(12) = 2.4,p = 0.016	t(12) = 1.77,p = 0.05
**right FFA**	t(15) = 1.48,p = 0.08	t(15) = 4.3,p<0.001	t(15) = 4.74,p<0.001	t(15) = 7.12,p<0.001	t(15) = 7.57,p<0.001
**left pSTS face area**	t(7) = 2.44,p = 0.02	t(7) = 2.54,p = 0.02	t(7) = 1.35,p = 0.11	t(7) = 2.47,p = 0.023	t(7) = 2.41,p = 0.026
**right pSTS face area**	t(11) = 2.03,p = 0.03	t(11) = 1.53,p = 0.08	t(11) = 2.63,p = 0.011	t(11) = 1.99,p = 0.035	t(11) = 1.31,p = 0.11
**left ATL face area**	t(6)<1	t(6) = 1,p = 0.17	t(6)<1	t(6) = 1.62,p = 0.07	t(6)<1
**right ATL face area**	t(10)<1	t(10)<1	t(10)<1	t(10)<1	t(10)<1
**left prefrontal face area**	t(7)<1	t(7)<1	t(7)<1	t(7)<1	t(7)<1
**right prefrontal face area**	t(12)<1	t(12) = 1.16,p = 0.13	t(12) = 1.32,p = 0.10	t(12) = 1.9,p = 0.04	t(12) = 1.38,p = 0.09
**left amygdala face area**	t(7)<1	t(7) = 1.31,p = 0.11	t(7) = 2.39,p = 0.02	t(7) = 2.07,p = 0.04	t(7) = 2.76,p = 0.014
**right amygdala face area**	t(7)<1	t(7) = 1.02,p = 0.16	t(7) = 1.69,p = 0.067	t(7) = 1.1,p = 0.15	t(7) = 1.74,p = 0.06
**left COS object area**	t(8)<1	t(8)<1	t(8)<1	t(8)<1	t(8)<1
**right COS object area**	t(11)<1	t(11)<1	t(11)<1	t(11) = 1.28,p = 0.11	t(11) = 1.26,p = 0.11

ROI sizes: 10 voxels (80 mm^3^), 20 voxels (160 mm^3^), 30 voxels (240 mm^3^), 40 voxels (320 mm^3^) and 50 voxels (400 mm^3^).

Our ROI analyses have shown that the FFA was the only region where face identities could be discriminated based on multivoxel patterns. Interestingly, face identity discrimination was at chance level in the ATL face-area—the region that was suggested to be important for face recognition [20,27,28]. Thus, to compliment the ROI analysis, we conducted search-light analysis [19] in the anterior temporal lobe. Due to limited brain coverage (coronal orientation, high-resolution), no search-light analysis was conducted for other regions. The search-light analysis was conducted using two schemes: in one scheme (the main analysis) the search-lights were overlapping, each voxel participated in many classifications and the results for each voxel were averaged; in an additional scheme (the control analysis), the search-lights were not overlapping and each voxel participated in one search-light only (see Methods). The results of classification in the anterior temporal using both schemes did not reveal any clusters, where the two famous identities could be discriminated above chance level (p<0.05, FDR corrected). It is noteworthy that the areas in the ATL where previous studies did reveal decoding of face identity [11,14,19] were more anterior (approximately more anterior MNI Y coordinate = 0) and were not covered in our study. We therefore do not claim that face identity is not represented in the ATL, but that it was not represented in the more limited brain regions covered by our high-resolution experiment where a face-selective area is found.

Finally, the analyses conducted for face identities were also performed for the two cup types. First, we tested whether, based on average fMRI signal, it is possible to discriminate between the two cups. The results are shown in Fig. 6A. No significant difference in activation between the two identities was found in any of the regions (paired, one-tailed t-test: left COS: t(8) = 2.56, p = 0.033; all other regions t<1). To decode cup type, we employed MVPA. The results are shown in Fig. 6B. In the left STS and left prefrontal cortex, the prediction of cup type was above chance level but did not reach significance after correction for multiple comparison: left STS (t(7) = 2.36, p = 0.025); right prefrontal (t(12) = 1.83, p = 0.045). In other regions, the prediction rate did not differ from chance: left FFA (t(12) = 1.69, p = 0.057); right amygdala (t(7) = 1.37, p = 0.1); right FFA (t(15) = 1.25, p = 0.11); all other regions t<1.

**Fig 7:**
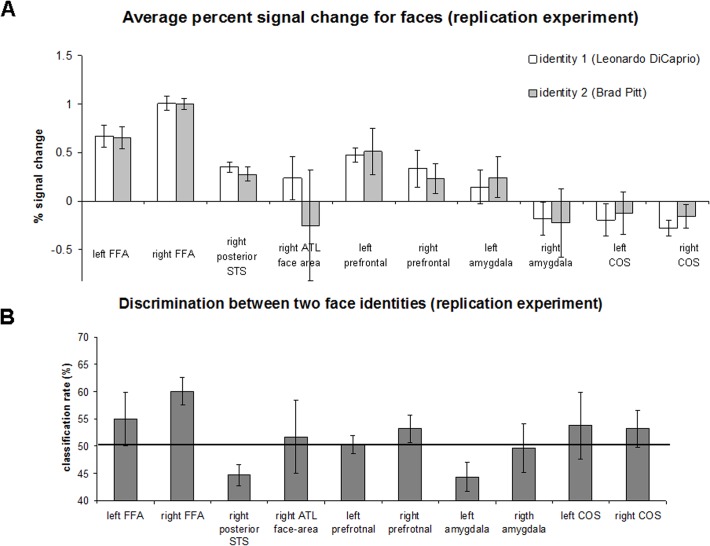
Region of Interest-based face identity discrimination analysis (replication experiment; Leonardo DiCaprio and Brad Pitt identities). (A) Average percent signal change for two face identities in the different face-selective areas and non-face selective collateral sulcus area. Error bars denote standard error of the mean. (B) Classification rates between face identities in face and non-face selective regions. The black line indicates a chance level of 50%. The error bars denote the standard error of the mean.

**Fig 8:**
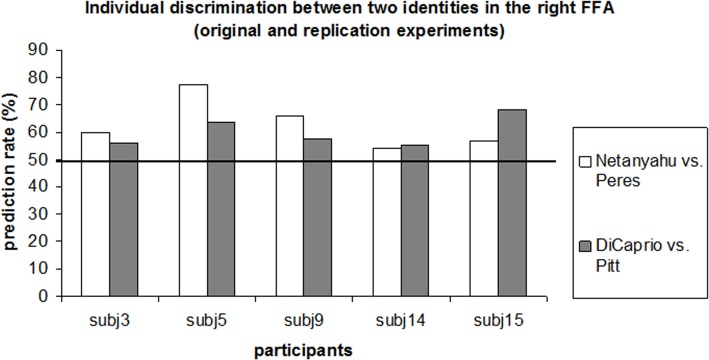
Comparison of individual classification rates of identity discrimination in the right FFA for the original (Benjamin Netanyahu and Shimon Peres) experiment and the replication (Leonardo DiCaprio and Brad Pitt).

**Replication experiment with a new set of famous faces.** The original experiment had two potential limitations related to generalization of the findings. First, the experiment used the same face identities for localization of the face-selective regions and for the face identity discrimination analysis; accordingly, it can be claimed that our discrimination results were specific for the face-selective voxels that were most sensitive to two specific identities. Second, the original experiment included only two face identities. To address both of these shortcomings, we ran the following experiment: a) the face-selective regions were localized using an independent functional localizer with various unfamiliar face and object stimuli (independent set of stimuli); and b) discrimination analysis was conducted for two additional famous face identities (Leonardo DiCaprio and Brad Pitt). The goal of the experiment was to test whether the successful decoding of the famous faces in the right FFA can be replicated using two novel identities. Five participants who participated in the original experiment participated in this experiment. Average fMRI signal and decoding results are shown in Fig. 7. Only ROIs localized in at least three participants are shown. The average fMRI signal for both face identities was similar. Critically, corroborating our findings of the original experiment, the average classification rate between two identities in the right FFA was the high, reaching the level of 60%. Despite the small sample size (N = 5), prediction in the right FFA was significantly beyond chance level (t(4) = 3.96, p = 0.008). In addition, in Fig. 8 we show individual classification rates between face identities in the original and the replication experiment. Critically, in all participants the prediction rate was beyond chance level (50%) for both face pairs. Some prediction rate variability between experiments could be explained by the temporal difference between experiments (about 3.5 years) and potential difference in semantic information associated with two categories (i.e., politicians vs. actors). Taken together the results of the replication experiment demonstrate that the two famous face identities could be discriminated in the right FFA, which was localized using independent functional localization procedure.

## Discussion

The goal of the current study was to systematically explore the role of face-selective areas in recognition of famous faces. To that end, we presented several different images of two famous identities and used MVPA to discover which areas can discriminate between the two famous identities. The analysis was conducted for two different pairs of famous identities. The key finding was that famous face identity can be decoded significantly above chance level in the right fusiform face area (FFA). This result corroborates the importance of the FFA in face recognition.

The role of the FFA in face recognition has been advocated for a long time [62]. Consistent with this view, the FFA has been shown to exhibit properties essential for face recognition, including holistic face processing [60,61,63–66], partial view and mirror invariance [35,52,67,68], and correlation with measures of face discrimination and recognition [12,15,69], but see [16]. Notably, the most direct measure to test whether a region is responsible for face identity processing is to show that—based on the neural signal of that region—two identities can be discriminated [70]. Interestingly, while early pattern classification studies failed to find evidence in support of the role of the FFA in face recognition [17,19], three recent studies demonstrate that unfamiliar identities could be discriminated across changes in facial expressions [14], face view [11] and features/configural changes [13]. Our results corroborate these findings by showing that different images of two famous face identities can be successfully discriminated in the fusiform face area (FFA) (Fig. 3B). The prediction rate in the right FFA was consistently above chance level across participants (Fig. 4) and across ROIs of different size (Fig. 5), suggesting the reliability of the effect. In addition, we replicated the result using two additional pairs of famous identities. It should be noted that since our design included only famous faces, we cannot estimate the extent to which rich semantic information that was part of famous stimuli we used contributed to the success of face discrimination. While based on results of early studies amount of semantic information in the FFA was minimal [71] or even absent [13], one recent study that used a more sensitive cluster analysis technique [72] was able to reveal semantic categories within the FFA. It is noteworthy that the semantic categories Çukur and colleagues have revealed were unrelated to faces. Thus, it is an open question whether semantic information about faces is also governed according to the same rules. In any case, in order to estimate the role of facial semantic information in the FFA, future studies will need to compare discrimination across both unfamiliar and famous identities within the same experiment. Finally, it should be noted that the view angle of the faces that we used was relatively small (up to ∼10° of view angle rotation). This was a deliberate decision as the FFA is known to be relatively view-selective [35,52] and was shown not to generalize across large angle rotations [17]. Notably, many previous studies that explored identity discrimination used images of faces with a single view only [12,14–16,19]. Now, as we established that famous faces with minimal view angle change can be discriminated, the next stage is to test discrimination across larger view angle changes.

A potential caveat in studies that discriminate between high-level visual categories in general and faces in particular is that neural discrimination can be based on low-level image properties [65,73]. Several steps were taken to address this point and ensure that the successful face discrimination was not based on low-level image properties [19,74] but rather involved face-processing mechanisms. First, the two face identities that were selected for the study were both males of similar age; they were grey-haired, and their faces did not differ by any distinctive markers, such as moustaches, beards or glasses [60,61] (Fig. 2A). Second, each of the two identities was represented by 8 different images, which were the pictures taken on different occasions mostly from the front view (up to ∼10° of view angle rotation). Third, luminance and contrast were adjusted between the images (see Materials and Methods). Fourth, to ensure that there was no low-level systematic difference between the two sets of images, we conducted image similarity analysis to show that images of the different identities were no more different than images of the same identity. Finally, there was no significant above chance discrimination between the two types of cups in the right FFA, although pixel-correlation analysis showed that the different cup types were different in terms of low-level features. Taken together, we suggest that discrimination between the two face identities is not likely to reflect discrimination based on low-level information but rather reflects face discrimination.

Interestingly, while the two famous face identities could be discriminated in the FFA, no discrimination was achieved in the anterior temporal lobe (ATL). The ATL is a large region that has been implicated in many functions, including social and emotional processing as well as semantic memory [38,75]. Small subparts of the ATL exhibit a face-selective response [28], but localization of these clusters is usually relatively unreliable due to severe magnetic susceptibility (e.g., [4,5,26]). In addition, several studies demonstrated that BOLD activity in the ATL contains information about face identity [11,13–15,19]. However, it is not clear whether these ATL clusters that showed identity decoding were face-selective [11,13–15,19]. Thus, the question of whether face identity is processed in the face-selective ATL is still unresolved. These findings in humans are inconsistent with the very clear representation of individual identities in the monkey most anterior face-selective patch (AM) [27], which has been considered the monkey homologue of the human face-selective ATL area [31].

Our study focused explicitly on an ATL face-selective area that was reliably localized using a recently proposed coronal scanning optimization method [9]. We found that face identities could not be discriminated in the ATL face-area (and also in adjacent non-face-selective voxels). To some extent, this result is even more surprising given that we used famous faces and the ATL is known to be a locus of semantic processing [76]. While it is possible that the ATL face-area indeed does not process face identity, it is also possible the "null result" stems from an insufficient sensitivity of the analysis or the still lower signal-to-noise ratio (SNR) in this area (relative to other face-selective areas), despite attempts to improve the signal by the coronal scanning [9]. Thus, whether the ATL face-area is essential for face recognition will require further investigation. Notably, our results do not contradict previous studies that found identity information in the ATL. That is, while the method we used mitigated susceptibility effects, it was also limited in terms of brain coverage. In particular, the anterior part of the ATL (approximately more anterior MNI Y coordinate = 0) has not been covered by our slice orientation. Yet, identity information has been mostly found in these more anterior parts of the ATL [11,14,15,19] (see also: [20]). One study that reported identity information in more posterior parts of the ATL [13] used two types of ATL clusters, including those clusters more adjacent to the FFA (MNI Y coordinate = -20/30)[9]—and may therefore reflect or partly reflect the FFA successful decoding rather than the ATL. Taken together, these methodological differences explain discrepancy in identity decoding.

The primary goal of the current study was to conduct a systematic exploration of the face-selective network with regard to discriminating face identity. Apart from a successful above chance discrimination in the right FFA, we also found a high prediction rate in the left FFA and left posterior superior temporal sulcus (pSTS), but the result did not reach significance after multiple comparison correction. Other regions that have not been explored before such as the face-selective area in the amygdala or the pre-frontal cortex did not show evidence of identity representation in our study. It is possible that the more abstract and multi-modal representation of faces in these areas could not be decoded with the current design that we used. For example, the choice of two faces from the same semantic categories (similar age, similar occupation, both famous) might have impeded our ability to reveal different neural codes to these two faces in these anterior areas. To test sensitivity to semantic-biographical information one may want to decode famous faces who at least have different occupations. In general, while the amygdala is believed to support emotional aspects of face-processing [77], the role of the prefrontal face-region in face processing is unclear to date[78]. For example, the region was more sensitive to dynamic than to static faces [79], was sensitive to the presence of eyes [10], was selective to the human body and its parts [10,79] and was possibly sensitive to working memory load [80]. Thus these previous results do not permit to draw a coherent picture. An additional possibility that should be considered is that the signals elicited by two identities were too similar to be detected by an fMRI—the method that has relatively low SNR [81]. To this extent identity decoding using single-cell recording in macaque monkeys might provide more information [82]. With respect to earlier visual areas, because we used high resolution, our coronal scanning did not include the occipital cortex and we could not examine the decoding level of the more posterior face and non-face areas (OFA and Lateral Occipital). The lack of Lateral Occipital coverage may explain why we did not find regions that discriminate between the two types of cup [42].

In conclusion, in the current study, we explored face discrimination of famous identities in the face-selective network of regions and in the adjacent non-face-selective cortex. Our key finding was that famous face identity could be decoded above chance level in the FFA face-selective region but not in other regions of the face-selective network. This result corroborates the important role played by the FFA in face recognition.
